# Intracellular ATP levels influence cell fates in *Dictyostelium discoideum* differentiation

**DOI:** 10.1111/gtc.12763

**Published:** 2020-03-13

**Authors:** Haruka Hiraoka, Tadashi Nakano, Satoshi Kuwana, Masashi Fukuzawa, Yasuhiro Hirano, Masahiro Ueda, Tokuko Haraguchi, Yasushi Hiraoka

**Affiliations:** ^1^ Graduate School of Frontier Biosciences Osaka University Suita Japan; ^2^ Institute for Datability Science Osaka University Suita Japan; ^3^ The United Graduate School of Agricultural Science Iwate University Morioka Japan; ^4^ Faculty of Agriculture and Life Science Hirosaki University Hirosaki Japan; ^5^ Center for Biosystems Dynamics Research (BDR), RIKEN Suita Japan; ^6^ Advanced ICT Research Institute Kobe National Institute of Information and Communications Technology Kobe Japan

**Keywords:** ATeam, ATP, ATP sensor probe, cell differentiation, glycolysis, live‐cell imaging, MaLion, metabolism, mitochondria, TCA cycle

## Abstract

Multicellular organisms contain various differentiated cells. Fate determination of these cells remains a fundamental issue. The cellular slime mold *Dictyostelium discoideum* is a useful model organism for studying differentiation; it proliferates as single cells in nutrient‐rich conditions, which aggregate into a multicellular body upon starvation, subsequently differentiating into stalk cells or spores. The fates of these cells can be predicted in the vegetative phase: Cells expressing higher and lower levels of *omt12* differentiate into stalk cells and spores, respectively. However, *omt12* is merely a marker gene and changes in its expression do not influence the cell fate, and determinant factors remain unknown. In this study, we analyzed cell fate determinants in the stalk‐destined and spore‐destined cells that were sorted based on *omt12* expression. Luciferase assay demonstrated higher levels of intracellular ATP in the stalk‐destined cells than in the spore‐destined cells. Live‐cell observation during development using ATP sensor probes revealed that cells with higher ATP levels differentiated into stalk cells. Furthermore, reducing the ATP level by treating with an inhibitor of ATP production changed the differentiation fates of the stalk‐destined cells to spores. These results suggest that intracellular ATP levels influence cell fates in *D. discoideum* differentiation.

## Graphical abstract

We analyzed differentiation fate determinants in the cellular slime mold *Dictyostelium discoideum*. Vegetable cells were sorted by cell sorter to collect stalk‐destined and spore‐destined cells, and ATP levels in those cells were visualized during development. Our results suggest that intracellular ATP levels act as an intrinsic cell fate determinant in *D. discoideum* differentiation.

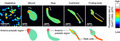

## 1 INTRODUCTION

Multicellular organisms consist of a variety of differentiated cells, and their differentiation processes must be tightly regulated to ensure their proper functions; errors that occur during the differentiation process may induce fatal defects in organisms. Thus, understanding the regulatory mechanisms that determine cell lineages is a fundamental question for the fields of biology and medicine (Avior, Sagi, & Benvenisty, 2016; Castanon & González‐Gaitán, 2011; Zakrzewski, Dobrzynski, Szymonowicz, & Rybak, 2019). At the beginning of the cell differentiation process, different cell types appear stochastically within a genetically identical cell population, which is known as the “salt and pepper” model and has been observed in various organisms, such as nematode worms, flies and mice (Chazaud, Yamanaka, Pawson, & Rossant, 2006; Miller, Seymour, King, & Herman, 2008; Schnabel et al., 2006). In such stochastic differentiation, non‐genetic cellular heterogeneity, which arises from fluctuations of intrinsic and extrinsic factors, appears to be a key factor in the determination of cell fates. Intercellular variations in gene expression, metabolism and responses to cellular signals have been proposed to be intrinsic factors that affect cell fate (Elowitz, Levine, Siggia, & Swain, 2002; Evers et al., 2019; Raser & O’Shea, 2004; Yamanaka & Blau, 2010). Among these potential factors, metabolism appears to play a significant role in cell fate decisions. Increasing evidence has indicated that the activity levels of the mitochondria, important organelles associated with metabolism, play a role in the differentiation of human cells (Buck et al., 2016; Khacho et al., 2016). However, the critical factors that determine cell fate remain unknown.

**Figure 1:**
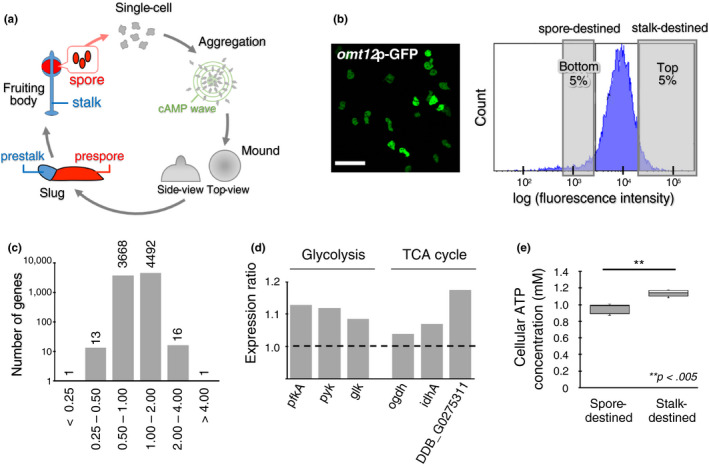
Gene expression analysis of stalk‐destined and spore‐destined cells. (a) Schematic illustration of the developmental process for *Dictyostelium discoideum*. A slug consists of prestalk cells in the anterior region (blue) and prespore cells in the posterior region (red). Prestalk cells differentiate into stalk cells, and prespore cells differentiate into spore cells of a fruiting body. (b) A typical image of vegetative cells expressing the *omt12*p‐GFP construct used for cell sorting (left) and typical distribution of the fluorescence intensities of *omt12*p‐GFP cells as measured by flow cytometry (right). The top 5% and bottom 5% of *omt12*p‐GFP‐expressing cells were collected as the stalk‐destined and spore‐destined cells, respectively, and used for further analysis. Scale bar, 50 µm. In this experiment, the *omt12*p‐GFP‐expressing strain, generated using the pDd plasmid vector described in Kuwana et al. (2016), was used. (c) Analysis of RNA‐seq results. The ratio of the expression level of a particular gene in stalk‐destined cells to that in spore‐destined cells (stalk/spore ratio) was calculated for 8,191 genes and averaged across 3 trials. The number of genes in a logarithmic scale was plotted as a histogram of the ratio. The number at the top of each column indicates the number of genes belonging to each bin. (d) The stalk/spore ratios for genes involved in major energy production. The left 3 and right 3 columns indicate the ratios of genes involved in glycolysis and the TCA cycle, respectively. (e) Cellular ATP concentrations as measured by luciferase assay. The *omt12*p‐GFP‐expressing strain was used as in (b). Results are presented as a box‐and‐whisker plot: The box indicates the median and the upper and lower quartiles; the whisker indicates the range. The number of measurements: *n* = 12 (spore‐destined) and *n* = 10 (stalk‐destined). **p < .005

The cellular slime mold *Dictyostelium discoideum* is an amoebozoa and represents a good model organism for studying relationships between cellular heterogeneity and cell differentiation during the development of multicellular organisms. Amoeboid cells continue to proliferate under nutrient‐rich conditions (vegetative phase). Upon starvation, amoeboid cells initiate the process of multicellular development, differentiating into 2 major cell types: stalk cells and spore cells (Figure 1a). In the early stages of development, amoeboid cells move collectively toward extracellular cAMP oscillations, originated from an aggregation center, to form a multicellular mound. Cells that enter the mound phase begin to differentiate into stalk or spore progenitor cells, called prestalk and prespore cells, respectively, which arise stochastically in a salt and pepper fashion. Prestalk cells are sorted to the top side of the mound, forming the tip region, which later forms the anterior region of the migrating body (slug), whereas prespore cells constitute the posterior region of the slug. In the process of fruiting body formation, prestalk cells differentiate into stalk cells, penetrating into the prespore region of the slug. Spore cells generating progenies are moved to the top of the fruiting body through the support of stalk cells (Maeda, Inouye, & Takeuchi, 1997).

In the developmental process of *D. discoideum*, non‐genetic cellular heterogeneities, which are generated by differences in intracellular calcium concentrations, cell cycles and metabolism levels, have been suggested to play significant roles in cell fate decisions (Baskar, Chhabra, Mascarenhas, & Nanjundiah, 2000; Cubbit, Firtel, Fischer, Jaffe, & Miller, 1995; Katz & Bourguignon, 1974; Leach, Ashworth, & Garrod, 1973; Maeda, 2005, 2011; Maeda, Ohmori, Abe, Abe, & Amagai, 1989; Saran, Azhar, Manogaran, Pande, & Nanjundiah, 1994; Tasaka & Takeuchi, 1981; Thompson & Kay, 2000; Zada‐Hames & Ashworth, 1978). Similar to humans and other higher eukaryotes, mitochondrial activity appears to be crucial when switching from growth to differentiation in the cellular slime mold. Furthermore, previous studies have reported that cell differentiation can be regulated by the presence or absence of glucose, which is an essential component of the glycolysis pathway, in the culture medium (Leach et al., 1973; Tasaka & Takeuchi, 1981; Thompson & Kay, 2000). These factors generating heterogeneities reflect cell conditions in the vegetative phase, implying that cell lineages are determined prior to differentiation according to the heterogeneities present in the vegetative phase.

In fact, a recent study has shown that cell fates in *D. discoideum* cells are predetermined in the vegetative phase, prior to multicellular formation, and can be predicted by expression levels of the *omt12* gene (Kuwana, Senoo, Sawai, & Fukuzawa, 2016). Vegetative cells expressing high levels of *omt12*, which are termed as pstV^A^ cells in Kuwana et al. (2016), are destined to differentiate into stalk cells (designated as “stalk‐destined” cells, hereafter), whereas vegetative cells expressing low levels of *omt12* are destined to differentiate into spore cells (designated as “spore‐destined” cells, hereafter). However, *omt12* is merely a marker gene, and changes in its expression levels do not influence the cell fates (Kuwana et al., 2016). The identities of the factors that drive differentiation of stalk‐destined cells (expressing high *omt12* levels) and spore‐destined cells (expressing low *omt12* levels) remain unknown.

To address this question, we first carried out RNA sequencing (RNA‐seq) analyses to examine differences in gene expression levels between stalk‐destined and spore‐destined cells sorted by flow cytometry based on *omt12* expression levels. The subsequent RNA‐seq analysis and luciferase assay suggested that differences in ATP levels may be a factor that affects differentiation. We next monitored intracellular ATP levels during differentiation, using ATP sensor probes that we optimized for use in *Dictyostelium*. We also investigated roles of ATP in *D. discoideum* differentiation by using specific inhibitors of ATP production.

## 2 RESULTS

### 2.1 Undifferentiated amoeba cells with high *omt12* expression have high ATP levels

To identify the intrinsic factors that determine cell fates, stalk‐destined and spore‐destined cells were separately collected in the vegetative phase, using a cell sorter, based on the fluorescence intensity of green fluorescent protein (GFP) expressed under the control of the *omt12* gene promotor (*omt12*p‐GFP), as described previously (Kuwana et al., 2016). The top 5% of cells with high *omt12*p‐GFP expression levels were collected as stalk‐destined cells, and the bottom 5% were collected as spore‐destined cells (Figure 1b and Figure S1). The mRNA transcripts contained in each cell population were analyzed by RNA‐seq analysis to comprehensively determine the expression levels of all genes. RNA‐seq data of 8,191 genes were selected for subsequent analysis as reliable data: Genes with low expression levels (count values) and with large variations in three trials were excluded as unreliable data (see Section 4 for details of thresholding). The results of the RNA‐seq analysis revealed that 31 out of the 8,191 genes showed differential expression levels larger than twofold differences between the two cell types (Figure 1c, Table S1 and Data set S1). Of these 31 genes, 14 showed higher expression levels in spore‐destined cells and 17 showed higher expression levels in stalk‐destined cells (Figure 1c and Table S1); the functions of most of these genes were unknown.

Because metabolism has been speculated to be associated with cell fate determination, in both *D. discoideum* (Kimura, Kuwayama, Amagai, & Maeda, 2010; Leach et al., 1973; Matsuyama & Maeda, 1995; Tasaka & Takeuchi, 1981; Thompson & Kay, 2000) and mammalian cells (Cho et al., 2006; Takubo et al., 2013), we focused on genes associated with metabolic pathways. Out of the 8,191 genes, 304 genes were selected as metabolism‐related genes, based on annotations in the *Dictyostelium* database (dictyBase; http://dictybase.org) that contained the words “metabolic process,” “glycolytic process” and “tricarboxylic acid (TCA) cycle” as glycolysis and TCA cycle are known to be primary metabolic pathways. The expression levels of these genes were compared between the stalk‐destined and spore‐destined cells, using results from RNA‐seq analysis. The results demonstrated that 210 genes, corresponding with approximately 70% of the selected 304 metabolism‐related genes, showed higher expression levels in stalk‐destined cells than in spore‐destined cells (Data set S2). Interestingly, this group included 6 genes that encode limiting enzymes associated with glycolysis and the TCA cycle (Figure 1d). Although the differences in the expression levels of these 6 genes were minor (Figure 1d and Table S2), this result suggested that a compounding effect resulting from the increased expression levels of these genes may increase the metabolic activities in stalk‐destined cells.

Because metabolic activity correlates with ATP levels (Depaoli et al., 2018), we quantified the cellular ATP concentrations by a luciferase assay in extracts from stalk‐destined and spore‐destined cells. The stalk‐destined (expressing high *omt12*p‐GFP levels) and spore‐destined (expressing low *omt12*p‐GFP levels) cells were sorted according to *omt12*p‐GFP levels. The results showed that the cellular ATP concentrations of stalk‐destined cells were higher than those of spore‐destined cells (Figure 1e), suggesting that metabolic activity increases in stalk‐destined cells during the vegetative phase. Because the process of cell sorting might artificially alter the metabolic activities of cells, we further confirmed the correlation between *omt12* expression levels and ATP levels in cells using microscopic measurements carried out in individual cells without cell sorting. For this experiment, we established a *D. discoideum* strain that co‐expresses *omt12*p‐mCherry and the ATP sensor fluorescent probe DicMaLionG (generated based on MaLionG with codons optimized for *D. discoideum*; see Section 4 for details of DicMaLionG construction). It has been reported that dissociation constant (Kd) of MaLionG is determined as 1.1 mM (Arai et al., 2018) and that intracellular ATP concentrations in *D. discoideum* are approximately 1 mM (Roos, Scheidegger, & Gerish, 1977). Thus, we used DicMaLionG as an appropriate ATP sensor probe to detect intracellular ATP levels in *D. discoideum*. Furthermore, the fluorescence intensity of DicMaLionG decreased when cells were treated with oligomycin, an inhibitor of ATP synthase, confirming that this probe was functional for the detection of ATP levels in *D. discoideum* (Figure S2a,b; Movie S1). Observations of the *omt12*p‐mCherry/DicMaLionG double‐expressing strain revealed that *omt12*p‐mCherry expression levels were correlated with ATP levels (fluorescence intensity of DicMaLionG) at the single‐cell level under physiological conditions (Figure S3a). In contrast, expression levels of *act15*p‐mRFPmars, which is expressed under a constitutive *act15* promoter as a control, showed no correlation with ATP levels (Figure S3b). This result demonstrated that stalk‐destined cells expressing high levels of *omt12*p‐mCherry had higher ATP levels in the vegetative phase, probably because of the compounding effects of slightly higher expression levels of multiple metabolism‐related genes.

### 2.2 ATP‐rich cells differentiate into stalk cells during development

**Figure 2:**
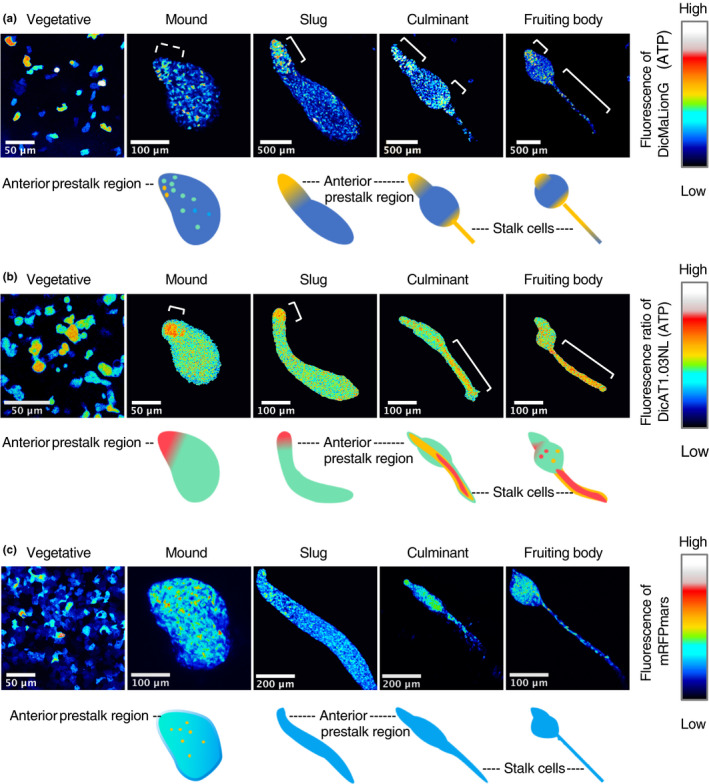
Imaging of ATP levels during *Dictyostelium discoideum* development. (a) Typical fluorescence images of DicMaLionG in each stage of development (upper panels) and schematics of the corresponding developmental stages (bottom). The DicMaLionG‐expressing strain was used. ATP levels are indicated by the color scale presented on the right. White lines indicate cells with high fluorescence intensity. Images were acquired by FV1000 and projected with the maximum intensity of *z*‐stack images using Fiji. Objective lenses used: 60× oil immersion lens for vegetative cells; 40× lens for the mound; and 20× lens for the slug, culminant and fruiting body. (b) Typical FRET ratio images of DicAT1.03Nl in each stage of development (upper panels) and schematics of the corresponding developmental stages (bottom). The DicAT1.03Nl‐expressing strain was used. ATP levels are indicated by the color scale presented on the right. White lines indicate cells with high fluorescence intensity. Images were acquired by LSM780 and projected with the maximum intensity of *z*‐stack images using Fiji. Objective lenses used: 60× oil immersion lens for vegetative cells; 40× lens for the mound; and 20× lens for the slug, culminant and fruiting body. (c) Typical fluorescence images of *act15*p‐mRFPmars in each stage of development (upper panels) and schematics of the corresponding developmental stages (bottom). The *act15*p‐mRFPmars‐expressing strain was used. Expression levels of *act15*p‐mRFPmars are indicated by the color scale presented on the right. Images were acquired by FV1000 and projected with the maximum intensity of *z*‐stack images using Fiji. Objective lenses used: 60× oil immersion lens for vegetative cells; 40× lens for mound; 20× lens for slug and culminant; and 40× lens for the fruiting body

We next examined the fates of cells with higher ATP levels. In this experiment, cells expressing DicMaLionG were observed at various developmental stages, including the mound, slug, culminant and fruiting body phases (Figure 2a). The results showed that cells with higher ATP levels were localized to the anterior prestalk region of the slug and the stalk region of the fruiting body (Figure 2a). To further confirm this result, a FRET‐type ATP sensor probe, DicAT1.03Nl, was used. DicAT1.03Nl was generated based on ATeam, AT1.03Nl (Kd = 1.4 mM; Tsuyama et al., 2013), using codons optimized for *Dictyostelium* (see Section 4 for details of DicAT1.03Nl construction). The fluorescence intensity of DicAT1.03Nl decreased when cells were treated with oligomycin, an inhibitor of ATP synthase, confirming that this probe was functional for the detection of ATP levels in *D. discoideum* (Figure S2c,d; Movie S2). DicAT1.03Nl revealed a similar localization pattern as that observed when using DicMaLionG (Figure 2b). In contrast, control cells expressing *act15*p‐mRFPmars showed a diffuse distribution throughout the multicellular body at various stages of development (Figure 2c). Furthermore, cellular ATP levels correlated with the expression levels of *omt12*p‐mCherry at various developmental stages, including the vegetative, aggregation, loose/tight mound and slug phases (Figure S4). These results implied that vegetative cells with high ATP levels moved to the anterior prestalk region in the slug and eventually differentiated into stalk cells in the fruiting body.

**Figure 3:**
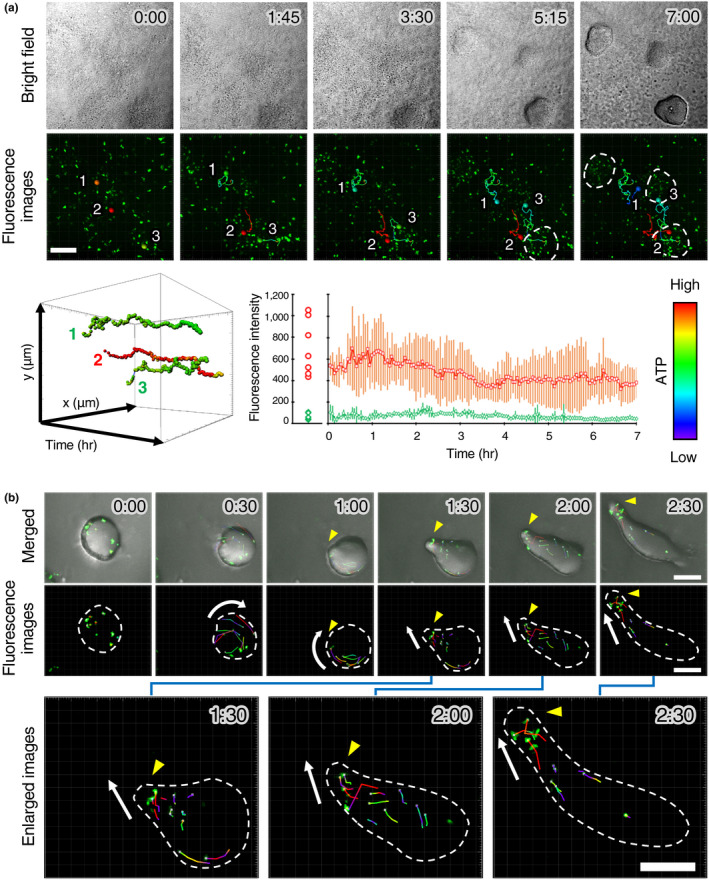
Tracking of target cells during development. (a) The tracking of single cells of interest during development from the vegetative phase to the mound phase. DicMaLionG‐expressing cells were mixed with wild‐type Ax2 cells (see Section 4 for details). Upper and middle panels indicate bright‐field and fluorescence images of DicMaLionG‐expressing cells, respectively. *Z*‐stack images were acquired every 3 min by Dragonfly200 using a 20× objective lens and projected with the maximum intensity using Fiji. The number in each image indicates the time as hr:min. Representative trajectories of three individual cells are shown in the fluorescence images; their colors represent the fluorescence intensities (ATP level) of individual cells according to the color scale at the right bottom. Dashed lines indicate the periphery of mounds. The 3D graph in the lower‐left panel shows the trajectories of these three cells over time; their colors represent the fluorescence intensities according to the color scale at the right bottom. The graph in the lower right panel plots the mean fluorescence intensities of all tracked cells with both high (red) and low ATP levels (green); the means and SDs are plotted over time (*n* = 7 and *n* = 5 for high and low ATP levels, respectively). The initial intensity of each cell is shown to the left of the graph. Scale bar, 100 µm. (b) The tracking of single cells of interest during development from the mound phase to the slug phase. DicMaLionG‐expressing cells were mixed with wild‐type Ax2 cells (see Section 4 for details). Upper panels indicate the merged images of bright‐field and fluorescence images, and middle panels indicate the fluorescence images, superimposed with trajectories over time. The colors of trajectories represent their intensities as described in (a). The number in each image indicates the time as hours:minutes. *Z*‐stack images were acquired every 3 min by Dragonfly200 using a 20× objective lens. Bottom panels indicate the enlarged images of the right 3 images of the middle panels. Yellow arrowheads indicate the tip regions, where the anterior stalk regions were formed. White arrows indicate the direction of cell movement. Scale bar, 100 µm

To confirm this assumption directly, we tracked individual ATP‐rich cells during differentiation from the vegetative phase to the slug phase, using the cell strain expressing DicMaLionG. This duration was a sufficiently long observation period for tracking cell fates because cells in the anterior prestalk region of a slug are known to subsequently differentiate to stalk cells in a fruiting body. ATP‐poor cells were also tracked as a control. The results demonstrated that both ATP‐rich and ATP‐poor cells maintained their ATP levels during the aggregation process from the vegetative phase to the mound phase (Figure 3a and Movie S3). At this point, both ATP‐rich and ATP‐poor cells were located throughout the mound with no clear separation. Next, the cells were tracked from the mound phase to the slug phase. ATP‐rich cells moved rotationally along the edge of the mound, repeatedly until the tip formation (see “0:30” to “1:00” of Figure 3b), and later moved toward the anterior stalk region of the slug (see red lines in “1:30” to “2:30” of Figure 3b and Movie S4). In contrast, ATP‐poor cells did not reach the anterior stalk region of the slug but instead remained in the middle region of the slug (see green and purple lines in “1:30” to “2:30” of Figure 3b and Movie S4). Taken together, these observations demonstrated that vegetative cells with high ATP levels moved toward the anterior stalk region in the slug while maintaining their high ATP levels, suggesting that the intracellular ATP levels of vegetative cells affect their cell fates.

### 2.3 Reducing ATP levels changes the differentiation fates of stalk‐destined cells to spores

**Figure 4:**
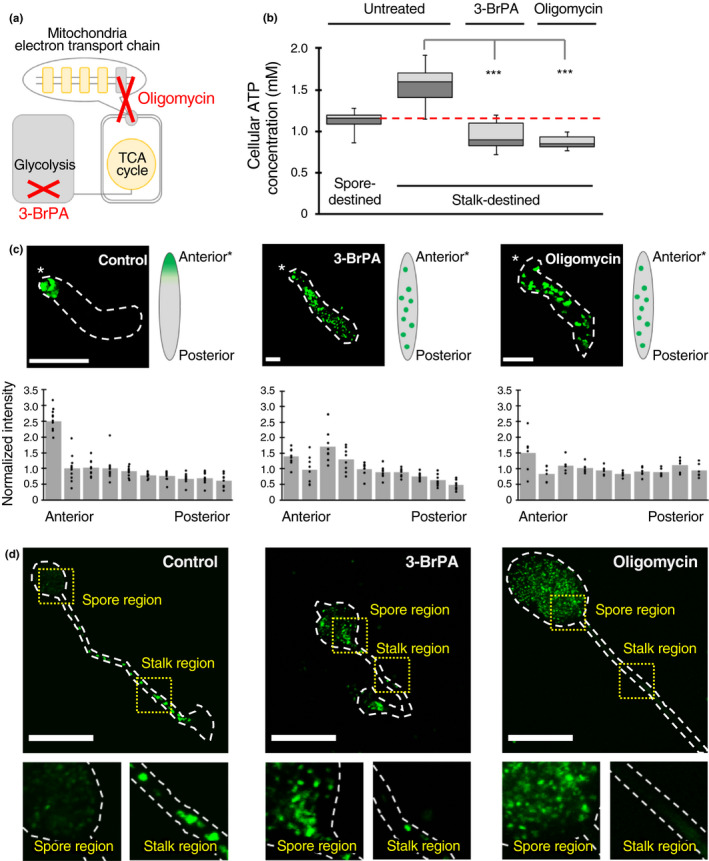
Effects of ATP production inhibitors on cell differentiation. (a) Schematic diagram showing the reaction sites where each inhibitor has an effect. 3‐BrPA inhibits the activity of the glycolysis limiting enzyme (hexokinase II), whereas oligomycin inhibits an ATP synthase required for ATP production in mitochondria. (b) Cellular ATP concentrations in untreated cells or cells treated with inhibitors (3‐BrPA or oligomycin) were measured by luciferase assay. *omt12*p‐GFP‐expressing cells (Kuwana et al., 2016) were sorted to obtain spore‐destined and stalk‐destined cell fractions (see Section 4 for details). The results are presented as a box‐and‐whisker plot: The box indicates the median and the upper and lower quartiles; the whisker indicates the range. The numbers of measurements were *n* = 27, 28, 15 and 13, from the left. The red dashed line represents the median value of cellular ATP concentrations in untreated spore‐destined cells. ***p < .001. (c) Fluorescence images of *omt12*p‐GFP in slugs. The stalk‐destined cells were mock‐treated (control, left panel) or treated with 3‐BrPA (middle panel) or oligomycin (right panel) and mixed at a 2:8 ratio with wild‐type Ax2 cells (not expressing GFP) for observing their cell fates. Dashed lines indicate the periphery of a slug. The schematic diagram on the right of each image shows an overview of the results obtained. Fluorescence images were acquired by FV1000 using a 20× objective lens and projected with the maximum intensity of z‐stack images by Fiji. The lower graphs show the GFP fluorescence intensity quantified in each section obtained by dividing the slug into 10 parts from the anterior to the posterior. Each section was divided to have the same area. The values were calculated as (intensity of each section)/(mean intensity of 10 sections). The number of samples tested, *n* = 11, 8 and 6 from the left. Scale bars, 50 μm. (d) Fluorescence images of *omt12*p‐GFP in fruiting bodies. The stalk‐destined cells were untreated (control, left panel) or treated with 3‐BrPA (middle panel) or oligomycin (right panel) and mixed at a 2:8 ratio with wild‐type Ax2 cells (not expressing GFP) for observing their cell fates. Dashed lines indicate the periphery of a fruiting body. The regions indicated by dotted squares are enlarged and displayed in the lower panels. Fluorescence images were acquired by FV1000 using a 20× objective lens and projected with the maximum intensity of *z*‐stack images by Fiji. Scale bars, 100 μm

To understand the role of ATP in cell differentiation, the intracellular ATP levels of vegetative cells were artificially reduced by inhibitor treatments, and the fates of treated cells were then monitored in a slug and a fruiting body. The following two inhibitors were used to reduce ATP concentrations: 3‐bromopyruvic acid (3‐BrPA), an inhibitor of hexokinase II enzyme which is a rate‐limiting enzyme in glycolysis (Geschwind, Ko, Torbenson, Magee, & Pedersen, 2002; Ko, Pedersen, & Geschwind, 2001); and oligomycin, an inhibitor of ATP synthase activity in mitochondria (Chappell & Greville, 1961; Kim & Berdanier, 1999; Figure 4a). We first tested conditions of inhibitor treatments that sufficiently reduce intracellular ATP concentrations of stalk‐destined cells to levels equal to or lower than the ATP concentrations in spore‐destined cells (Figure 4b). Under these conditions, stalk‐destined cells expressing *omt12*p‐GFP were treated with one of the two inhibitors to reduce intracellular ATP concentrations. These treated cells were mixed with non‐fluorescent wild‐type cells treated with solvent (mock‐treated) to ensure the progression of development. Then, we observed the fates of inhibitor‐treated stalk‐destined cells in a slug (Figure 4c). In the control, mock‐treated stalk‐destined cells were localized to the anterior prestalk region of the slug, as expected (Figure 4c, left). In contrast, stalk‐destined cells treated with 3‐BrPA were scattered throughout the slug, instead of being localized to the anterior prestalk region (Figure 4c, middle). Similarly, stalk‐destined cells treated with oligomycin were also scattered throughout the slug (Figure 4c, right). To quantify the localization of stalk‐destined cells in a slug, fluorescence intensity of *omt12*p‐GFP was measured in the slug divided into 10 sections from the anterior to the posterior. The results revealed that mock‐treated stalk‐destined cells showed strong GFP localization in the anterior region, whereas stalk‐destined cells treated with 3‐BrPA or oligomycin showed scattered localization throughout the slug (Figure 4c, bottom). Next, we observed the fates of inhibitor‐treated stalk‐destined cells in a fruiting body (Figure 4d). In the control, mock‐treated stalk‐destined cells were localized to the stalk region, but not to the spore region, as expected (Figure 4d, left). In stark contrast, stalk‐destined cells treated with 3‐BrPA or oligomycin were localized to the spore region, but not to the stalk region (Figure 4d, middle and right, respectively), suggesting that inhibitor‐treated stalk‐destined cells changed their differentiation fates to spore cells.

The two inhibitors tested in these experiments inhibit different metabolic pathways—3‐BrPA affecting glycolysis and oligomycin affecting mitochondrial ATP synthesis, but caused the same results. Because ATP production is the common goal of these two pathways, decreasing the production of ATP may be responsible for changing the differentiation fates of stalk‐destined cells. Taken together, these results suggest that ATP is an important factor associated with cell fate determination in *D. discoideum* differentiation.

## 3 DISCUSSION

Intercellular variation in metabolic activity is one of the non‐genetic cellular heterogeneities. In cellular slime molds, the activity of mitochondria, which produce ATP, has been proposed to be an important factor for differentiation. For example, it is known that the expression of genes encoded by mitochondrial DNA is necessary when switching from growth to differentiation (Inazu, Chae, & Maeda, 1999; Maeda, 2005), that increased mitochondrial respiratory activity leads to increased stalk differentiation (Kimura et al., 2010; Matsuyama & Maeda, 1995) and that mitochondrial morphology affects cell differentiation because the maturation of spore cells requires a component of the mitochondrial structure (Hirata, Amagai, Chae, Hirose, & Maeda, 2008; Maeda, 1971, 2005; Matsuyama & Maeda, 1998). Because mitochondria produce ATP (Cooper, 2000), the importance of mitochondria during differentiation supports our hypothesis that cellular ATP levels are important when determining the direction of cell fate differentiation in *Dictyostelium*.

Differentiation‐related factors present in vegetative cells of *Dictyostelium*, such as intracellular calcium concentrations and the cell cycle, can be affected by mitochondrial activity. High intracellular calcium concentrations have been shown to cause increased stalk differentiation in *Dictyostelium* (Baskar et al., 2000; Cubbit et al., 1995; Saran et al., 1994), and in most organisms, intracellular calcium concentrations are regulated by the flux of calcium ions stored in mitochondria or the endoplasmic reticulum (Berridge, Bootman, & Roderick, 2003; Stefani, Raffaello, Teardo, Szabo, & Rizzuto, 2011). The cell cycle stage has been proposed to determine the direction of cell differentiation fates (Maeda, 2005, 2011). The switching point from growth to differentiation is present in the cell cycle, and the expression of genes encoded by mitochondrial DNA is required for transition at this cell cycle point (Inazu et al., 1999; Maeda, 2005). However, our RNA‐seq analysis revealed no significant differences in the expression levels of calcium‐related genes or cell cycle‐related genes between stalk‐destined and spore‐destined cells (Tables S3, S4); thus, the effects of these factors on the ATP‐dependent cell fate decision remain unclear.

During the late developmental stages, prestalk cells are sorted to the tip region of a mound. The tip region has been reported to act as a cAMP source (Matsukuma & Durston, 1979), and prestalk cells move to cAMP faster than prespore cells (Abe, Early, Siegert, Weijer, & Williams, 1994; Early, Abe, & Williams, 1995; Traynor, Kessin, & Williams, 1992). In addition to this classical view, a recent study demonstrates that differences in preferences to cAMP chemotaxis and cell–cell contact between prestalk and prespore cells are important: cAMP chemotaxis is dominant in prestalk cells, and cell–cell contact is dominant in prespore cells (Fujimori, Nakajima, Shimada, & Sawai, 2019). These findings raise the possibility that prestalk cells possess increased cAMP responsiveness compared with prespore cells. cAMP molecules are synthesized from ATP molecules within each cell; thus, high ATP levels in stalk‐destined cells may increase the responsiveness of these cells to cAMP, accelerating initial movements toward the tip region. However, the actual effects of ATP on cAMP chemotaxis during *Dictyostelium* development have not yet been elucidated*.* To address why ATP‐rich prestalk cells are sorted to the tip region, further analysis is necessary.

In *Dictyostelium,* approximately 20% of total cells form the stalk, which is removed by apoptosis, whereas the remaining cells propagate as spores (Bonner & Dodd, 1962; Hayashi & Takeuchi, 1976). Our results imply that ATP‐rich cells preferentially differentiate into stalk cells, which will be removed from the population, whereas cells with low ATP levels are selected to survive as spores. Cells with high ATP levels are likely to maintain high levels of mitochondrial activity, which can increase damaged mitochondrial DNA generated by reactive oxygen species, by‐products of mitochondrial activity. Thus, ATP‐rich cells may be targeted for eventual removal by differentiation into stalk cells. This hypothesis is supported by evidence demonstrating that cells exhibiting damaged mitochondrial DNA tend to differentiate into stalk cells (Chida, Yamaguchi, Amagai, & Maeda, 2004).

Recent lines of evidence increasingly indicate a relationship between metabolic activity and cell differentiation in various organisms, including mice and humans (Berger et al., 2016; Buck et al., 2016; Cho et al., 2006; Khacho et al., 2016; Rodríguez‐Colman et al., 2017; Schell et al., 2017; Simsek et al., 2010; Yang, Shen, Reece, Chen, & Yang, 2016). The ratio of glycolysis and TCA cycle usage has been argued to affect cell fates at the growth/differentiation transition (Buck et al., 2016; Takubo et al., 2013), and the metabolites produced by each pathway may also affect cell fates (Khacho et al., 2016). Furthermore, in cancer cells, metabolic shifts between glycolysis and the TCA cycle have been reported to affect cell fates at the growth/differentiation transition (Hsu & Sabatini, 2008; Koppenol, Bounds, & Dang, 2011; Matoba et al., 2006). These findings suggest that crosstalk between metabolic activity and cell differentiation is widely conserved across various living organisms from cellular slime molds to humans. Moreover, increased mitochondrial biogenesis and ATP production have been reported during the early differentiation stages of human embryonic stem cells (Cho et al., 2006), suggesting a role for ATP in the differentiation of human cells. ATP may be a differentiation effector that is commonly used in all living organisms.

## 4 EXPERIMENTAL PROCEDURES

### 4.1 Plasmid constructs

The pDM vectors (Veltman, Akar, Bosgraaf, & Haastert, 2009) were used as the backbone vectors for generating the following DNA constructs. To generate the DNA plasmid (pDM358‐*omt12*p‐mCherry) expressing mCherry under the promoter of the *omt12* gene (*omt12*p‐mCherry) in *Dictyostelium* cells, the pDM304‐*omt12*p‐mCherry vector (a gift from Drs. Hashimura and Ueda, Osaka University) was digested with XhoI and SpeI restriction enzymes, and the resulting DNA fragment of the *omt12*p‐mCherry sequence was inserted into the pDM358 vector to render drug resistance to hygromycin. To generate the pDM304‐DicMaLionG vector encoding an ATP sensor probe that functions in *Dictyostelium*, codon optimization and DNA fragment synthesis were carried out by GenScript service (https://www.funakoshi.co.jp/contents/687) according to the information on *D. discoideum* codon usage (http://dictybase.org) and MaLionG sequences (Arai et al., 2018). The synthesized DNA fragment coding the optimized MaLionG sequence was cloned into the pDM304 vector (designated as pDM304‐DicMaLionG) digested with BglII and SpeI restriction enzymes. To generate the pDM304‐DicAT1.03Nl vector encoding a FRET‐based ATP sensor probe that functions in *Dictyostelium*, codon optimization and DNA fragment synthesis were carried out as described above, except that the sequence of an ATeam probe AT1.03Nl (Imamura et al., 2009; Tsuyama et al., 2013) was used instead of that of MaLionG.

### 4.2 *Dictyostelium discoideum* strains

The *D. discoideum* wild‐type strain Ax2 (in‐house strain) was used as the parental strain. The strain was cultured at 22°C in HL5 medium, supplemented with 10 µg/ml streptomycin/penicillin (SP) solution (168‐23191, FUJIFILM Wako Pure Chemical Corp.), to prevent contamination.

Cell strains constitutively expressing DicMaLionG or DicAT1.03Nl were generated by transfecting the pDM304‐DicMaLionG DNA plasmid or the pDM304‐DicAT1.03Nl DNA plasmid, respectively, into wild‐type Ax2 cells (in‐house strain), using electroporation; 20 µg/ml neomycin (074‐05963, FUJIFILM Wako Pure Chemical Corp.) was used for the selection of plasmid‐expressing strains. The resulting strains are referred to as the DicMaLionG‐expressing strain (used in Figures 2a, 3 and Figures S2a,b) and the DicAT1.03Nl‐expressing strain (used in Figure 2b and Figure S2c,d). pDM358‐*act15*p‐mRFPmars DNA plasmids (a gift from Mr Degawa and Dr Ueda, Osaka University) were transfected into wild‐type Ax2 cells by electroporation; 20 µg/ml hygromycin (084‐07681, FUJIFILM Wako Pure Chemical Corp.) was used for selection of *act15*p‐mRFPmars‐expressing strains (used in Figure 2c).

For the simultaneous imaging of *omt12* expression levels and ATP levels, pDM358‐*omt12*p‐mCherry DNA plasmids were transfected into the DicMaLionG‐expressing strain by electroporation; 20 µg/ml hygromycin (084‐07681, FUJIFILM Wako Pure Chemical Corp.) and 20 µg/ml neomycin were used for the selection of the *omt12*p‐mCherry/DicMaLionG double‐expressing strain (used in Figure S3a,S4). As a control strain, pDM358‐*act15*p‐mRFPmars DNA plasmids (a gift from Mr Degawa and Dr Ueda, Osaka University) were transfected into the DicMaLionG‐expressing strain by electroporation; 20 µg/ml hygromycin and 20 µg/ml neomycin were used for the selection of the *act15*p‐mRFPmars/DicMaLionG double‐expressing strain (used in Figure S3b).

Electroporation was carried out using an ECM830 electroporator (BTX) in all experiments (electric conditions: 500 V, 100 μs × 10 pulses, 1‐s interval). Clonal cells were selected on the basis of fluorescence intensity on a 96‐well plate (1860‐096, IWAKI Science Products) and used for this study.

### 4.3 Induction of cell differentiation

Cultured cells were washed twice with potassium phosphate buffer (KK2: 16.1 mM KH_2_PO_4_, 4.0 mM K_2_HPO_4_, pH 6.1) and suspended at a density of 4 × 10^7^ cells/ml in KK2 buffer. Then, 5‐µl droplets of cell suspensions (2 × 10^5^ cells) were spotted on agar plates to analyze the process of development. 1 ml of 1% Bacto Agar (214010, BD Biosciences) dissolved in milliQ was plated on a 35‐mm plastic dish as a platform of cell differentiation.

### 4.4 Cell sorting

For cell sorting based on *omt12*p‐GFP expression levels, the neomycin‐resistant *omt12*p‐GFP strain generated by Kuwana et al. (2016) was used (used in Figures 1, 4 and Figure S1). This strain was constructed based on the pDdGFP plasmid, encoding GFP under the control of the *omt12* gene promotor (Kuwana et al., 2016). Ax2 (wild‐type) cells were used as a negative control.

For sample preparations, the cells were first suspended at a density of 5 × 10^6^ cells/ml in HL5 medium. Then, 1 ml of the cell suspension was subjected to centrifugation to collect the cells into a pellet. The pellets were washed twice with KK2 buffer and resuspended in 0.8–1 ml of 0.3 mM EDTA in KK2 buffer to avoid cell–cell attachment. During this sample preparation process, cells were maintained on ice.

The cell suspension was applied to a FACSAria III flow cytometer (BD Biosciences) for cell sorting. FACS gating was set using the Ax2 cells in the order of FSC‐A/SSC‐A, FSC‐A/FSC‐W, SSC‐A/SSC‐W and GFP‐A/count. Cells with the top 5% GFP fluorescence intensity and those with the bottom 5% GFP fluorescence intensity were sorted as stalk‐destined and spore‐destined cells, respectively.

The sorted cells were collected in 2 ml of HL5 medium containing 30 μg/ml chloramphenicol (08027‐14, Nacalai Tesque) and then transferred into a 6‐cm plastic dish (3010‐060, IWAKI Science Products). The medium containing the sorting solution was replaced with fresh HL5 medium within 2 hr after sorting. These cells were cultured in the medium for 8–12 hr to recover from cell damage caused by the cell sorting procedure and were then used for subsequent experiments. The quality of cell sorting was evaluated by reanalyzing the fluorescence distribution of the sorted cells and quantifying the GFP expression levels by real‐time (RT) PCR as described below (Figure S1). The quality‐checked cell fractions were used for further analysis.

### 4.5 RNA extraction and RT‐PCR

Total RNA was extracted from the quality‐checked sorted cells using the RNeasy Plus Mini Kit (74134, QIAGEN), followed by the removal of contaminated genomic DNA using the RNase‐free DNase set (79254, QIAGEN). Then, 1 µg of the extracted total RNA was reversely transferred into cDNA using M‐MuLV Reverse Transcriptase (M0253S, New England BioLabs). RT‐PCR was carried out with THUNDERBIRD SYBR qPCR Mix (QPS‐201; TOYOBO) using the cDNAs as templates by Eco Real‐Time PCR system (Illumina). The PCR conditions were as follows: 1 cycle at 95°C for 1 min and 40 cycles at 95°C for 15 s, 55°C for 15 s and 72°C for 30 s. The primer sequences used for the quantification of GFP expression were 5′‐ATGTCTAAAGGAGAAGAACTTTTC‐3′ (FASMAC) as the forward primer and 5′‐TAAGTTTTCCGTATGTTGCATC‐3′ (Sigma‐Aldrich Inc.) as the reverse primer.

### 4.6 RNA‐seq analysis

The quality‐checked cell fractions of cell sorting were used for RNA‐seq analysis. Total RNA was extracted as described above. Comprehensive RNA‐seq analyses were carried out by the NGS service (http://ngs‐service.biken.osaka‐u.ac.jp/index.php/ja/pricing‐and‐ordering‐2/) of the Genome Information Research Center (Osaka University, Suita, Japan). Measurements were carried out in three biological replicates to obtain FPKM values and counts. Genes were excluded by two thresholding steps: first, genes with counts below 10 in either of the three experiments were excluded because these genes produce unreliable values for the stalk/spore ratio; second, genes with larger than twofold variations in the stalk/spore ratio across the three experiments were excluded to ensure reproducibility.

To analyze the differences in the gene expression levels between the stalk‐destined and spore‐destined cell, the genes of interest were selected based on the annotation in the *Dictyostelium* database (dictyBase; http://dictybase.org). Metabolism‐related genes were selected using the words “metabolic process,” “glycolytic process” and “TCA cycle” in the gene ontology section of the dictyBase. Calcium‐related genes were selected using the phrase “upregulated in the presence of calcium” in the “gene description” section of the dictyBase. Cell cycle‐related genes were selected by referring to previous studies on the relationship between cell cycle and differentiation (Maeda, 2005, 2011).

### 4.7 Luciferase assay for the quantification of cellular ATP levels

Cells were suspended in HL5 medium at a density of 2 × 10^5^ cells/ml. Then, 50 µl of the cell suspension (containing 1 × 10^4^ cells) and the same volume of CellTiter‐Glo2.0 assay solution (G9242; Promega) were added into each well of a 96‐well plate (3912; Corning) and mixed vigorously by shaking for 5 min at room temperature (25°C). The specimens were left undisturbed on a bench for 30 min at room temperature to allow the reaction to occur, and then the amount of luminescence was measured for 1 s using a luminometer (GloMax, Promega). Various concentrations of ATP solutions were prepared with ATP powder (01072‐11, Nacalai Tesque) for plotting a calibration curve. Cellular ATP levels were calculated from the amount of luminescence in the wells with the assumption that a cell is spherical with a diameter of 10 μm.

### 4.8 Fluorescence live‐cell imaging

Fluorescence live‐cell imaging was carried out by confocal microscopy as described below. Snapshot images were acquired with FV1000 (Olympus) using 20× (UPlanSApo 20×/0.75 NA, Olympus), 40× (UPlanSApo 40×/0.95 NA, Olympus) or 60× oil immersion (UPlanApoN 60×/1.42 NA, Olympus) objective lens. Diode lasers and HeNe lasers were used as sources for providing 473‐nm (GFP excitation) and 559‐nm (RFP excitation) wavelength lights, respectively. Time‐lapse images were acquired using Dragonfly200 (Andor) equipped with EMCCD (iXonUltra 888, Andor) cameras using 20× (Plan Apo 20×/0.75 NA, Nikon) and 60× oil immersion (Apo 60×/1.40 NA, Nikon) objective lenses. A solid‐state laser (ILE‐400, Andor) was used as the source for providing 488‐nm (GFP excitation) and 561‐nm (RFP excitation) wavelength lights. FRET images of DicAT1.03Nl cells were acquired by LSM780 (Zeiss) using 20× (Plan‐Apochromat 20×/0.8 NA, Zeiss), 40× water immersion (C‐Apochromat 40×/1.20 NA, Zeiss) and 60× oil immersion (Plan‐Apochromat 63×/1.40 NA, Zeiss) objective lenses. Multi Ar lasers were used for providing 458‐nm (CFP excitation) wavelength light. The FRET images represented the ratio intensities of YFP (ATP‐bound)/CFP (ATP‐free); they were obtained using “Ratio Plus,” a plugin of the Fiji software (ver. 2.0.0‐rc‐69/1.52p, https://fiji.sc (Schindelin et al., 2012)).

Sample setting for observing the developmental process of *D. discoideum* was carried out as described previously (Hashimura, Morimoto, Yasui, & Ueda, 2019). Briefly, a piece of agar plate used for assessing cell development was cut out and placed upside down on a liquid paraffin‐coated 35‐mm glass‐bottom dish (P35G‐1.5‐14‐C, MatTek). Wet papers were placed on the lid and around the glass part of the dish to prevent drying during time‐lapse imaging. *Z*‐stack images (5‐μm steps for 80–100 μm) were observed for acquiring the whole images of the 3D multicellular body.

To observe the vegetative cells, cells were plated on the glass‐bottom dish and the dish was filled with 2 ml of KK2 buffer after cell attachment.

### 4.9 Tracking the cells of interest during development

Cells were washed twice with KK2 buffer and suspended in the same buffer as described in Section 4.3. For tracking fluorescently labeled cells during the developmental process, 1%–5% of labeled cells were mixed with unlabeled cells. For tracking cells in early development (the vegetative phase to the mound phase), 20 μl of the mixed cell suspension at a density of 5 × 10^6^ cells/ml was added into each well of a 384‐well glass‐bottom plate (781091, Greiner Bio‐One Japan) to limit their movements to a small area. Then, 10 µl of excess solution was removed after cell attachment, and 40 μl of 2% low‐melting‐point agarose (15517‐022, Gibco‐BRL) in milliQ was layered on the cell suspension for flattening the 3D multicellular body. For tracking cells in late development (the mound phase to the slug phase), mixed cells were developed on the agar plate and a piece of agar was cut out and observed as described in the Sections 4.3 and 4.8.

Cell tracking analysis against this observation was carried out by the 3/4D Image Visualization and Analysis Software “Imaris” (ver.9.3.1; Bitplane AG; https://imaris.oxinst.com).

### 4.10 Treatment with inhibitors of ATP production

3‐Bromopyruvic acid (3‐BrPA; sc‐260854; Santa Cruz Biotechnology) or oligomycin (mixture of A, B and C isomers; O‐500, Alomone Labs), inhibitors of ATP production, was added to HL5 medium at the final concentrations of 1 mM and 2.5 μM, respectively. Cells were incubated at 22°C in HL5 medium containing each inhibitor for 24 hr, washed twice with KK2 buffer and then suspended in the same buffer for subsequent experiments.

### 4.11 Quantification and statistical analysis

The data shown in Figures 1e, 4b and Figure S1b are presented as box‐and‐whisker plots: The box indicates the median and the upper and lower quartiles; the whisker indicates the range. The precise number (*n*) of samples in each experiment is indicated in the figure legends. Statistical analyses were conducted using an unpaired, two‐tailed, Student's *t* test. Significant differences are presented as **p < .005 and ***p < .001.

The data shown in Figure 3a and Figure S2 are presented as the mean ± standard deviation (*SD*).

## CONFLICTS OF INTEREST

The authors declare no competing financial interests.

## Supporting information

Supplementary Material

Data set S1

Data set S2

Movie S1

Movie S2

Movie S3

Movie S4

## Data Availability

The processed RNA‐seq datasets analyzed in this study are available in Data set S1. Raw data of RNA‐seq are available at the DDBJ Sequence Read Archive (https://www.ddbj.nig.ac.jp/), with accession number DRA009715.
