# Supplementary material for: Intracellular ATP levels influence cell fates in Dictyostelium discoideum differentiation
Source: Genes Cells. 2020 Mar 13;25(5):312–26. doi: 10.1111/gtc.12763 (PMC7318147; doi:10.1111/gtc.12763)
Supplement: Supplementary file 1 — Supplementary Material [file GTC-25-312-s001.pdf]

Supporting Information

Intracellular ATP levels influence cell fates in *Dictyostelium discoideum* differentiation

*Haruka Hiraoka[1], Tadashi Nakano[2], Satoshi Kuwana[3], Masashi Fukuzawa[4], Yasuhiro Hirano[1], Masahiro Ueda[1,5], Tokuko Haraguchi[1,6], *Yasushi Hiraoka[1,6]

List of contents:

Table S1

Table S2

Table S3

Table S4

Figure S1

Figure S2

Figure S3

Figure S4

Legend for Movie S1

Legend for Movie S2

Legend for Movie S3

Legend for Movie S4

Legend for Data set S1

Legend for Data set S2

		Gene name	Protein name	Ratio
Higher in the stalk-destined cells	1	DDB_G0268640	hypothetical protein DDB_G0268640	5.030
	2	DDB_G0276219	hypothetical protein DDB_G0276219	3.102
	3	DDB_G0280703	elongation factor 1b-related protein	3.006
	4	DDB_G0290545	hypothetical protein DDB_G0290545	2.749
	5	DDB_G0276731	hypothetical protein DDB_G0276731	2.739
	6	abcC10	ABC transporter C family protein	2.685
	7	DDB_G0284277	hypothetical protein DDB_G0284277	2.435
	8	abcG10	ABC transporter G family protein	2.375
	9	DDB_G0272160	hypothetical protein DDB_G0272160	2.334
	10	cinB	esterase/lipase/thioesterase domain-containing protein	2.329
	11	DDB_G0275161	hypothetical protein DDB_G0275161	2.234
	12	DDB_G0272146	hypothetical protein DDB_G0272146	2.193
	13	DDB_G0276097	hypothetical protein DDB_G0276097	2.172
	14	cbpD1	calcium-binding protein	2.112
	15	DDB_G0288507	arylamine N-acetyltransferase family protein	2.045
	16	DDB_G0290975	alpha/beta hydrolase fold-3 domain-containing protein	2.026
	17	cafA	hypothetical protein DDB_G0277827	2.001
Higher in the spore-destined cells	1	DDB_G0288591	short-chain dehydrogenase/reductase family protein	0.497
	2	expl2	expansin-like protein	0.495
	3	DDB_G0271218	hypothetical protein DDB_G0271218	0.482
	4	DDB_G0280051	hypothetical protein DDB_G0280051	0.476
	5	DDB_G0267868	hypothetical protein DDB_G0267868	0.430
	6	DDB_G0280461	LISK family protein kinase	0.425
	7	DDB_G0281607	hypothetical protein DDB_G0281607	0.423
	8	cnrJ	carbohydrate-binding domain-containing protein	0.414
	9	DDB_G0269452	hypothetical protein DDB_G0269452	0.350
	10	DDB_G0287581	hypothetical protein DDB_G0287581	0.341
	11	celB	cellulase 270-11	0.317
	12	DDB_G0282715	hypothetical protein DDB_G0282715	0.313
	13	sigB	"peptidase M8, leishmanolysin family protein"	0.312
	14	DDB_G0280919	hypothetical protein DDB_G0280919	0.239

Table S1. Genes with differences larger than 2-fold

List of 31 genes with large differences of expression between stalk-destined and spore-destined cells: 17 genes with higher expression in the stalk-destined cells (listed in upper part) and 14 genes with higher expression in the spore-destined cells (listed in lower part). The “Ratio” values indicate the mean of the ratios of the expression levels of each gene in the stalk-destined cell to that in the spore-destined cell for three RNA-seq measurements.

Pathway	Gene name	Protein name	1st (FPKM)		2nd (FPKM)		3rd (FPKM)		Mean ratio	Fig.1D
			stalk-	spore-	stalk-	spore-	stalk-	spore-		
Glycolysis	pfkA	6-phosphofructokinase	126.99	108.11	137.68	133.22	164.56	140.01	1.128	✓
	pyk	pyruvate kinase	735.03	595.95	532.47	522.61	673.80	610.28	1.119	✓
	glk	glucokinase	35.98	34.39	29.40	26.46	43.99	40.05	1.085	✓
TCA cycle	ogdh	"2-oxoglutarate dehydrogenase, E1 subunit"	168.78	185.38	590.99	517.37	199.63	188.22	1.038	✓
	odhA	oxoglutarate dehydrogenase	66.84	53.88	93.41	78.99	69.21	58.94	1.199	
	idhA	isocitrate dehydrogenase (NAD+)	503.16	476.11	1065.15	955.41	501.19	483.59	1.069	✓
	idhB	isocitrate dehydrogenase (NAD+)	431.80	437.02	665.81	549.86	507.14	448.69	1.110	
	DDB_G0275311	"citrate synthase, mitochondrial"	1833.92	1496.73	2570.88	2267.00	2094.84	1797.53	1.175	✓
	cshA	citrate synthase	85.89	128.37	611.55	579.61	89.72	103.41	0.864	
	gltA	citrate synthase	47.40	42.65	72.51	63.27	37.09	34.02	1.116	

Table S2. Genes encoding limiting enzymes in the glycolysis and TCA cycle

List of 10 genes encoding limiting enzymes in the glycolysis and TCA cycle. As several genes involved in the TCA cycle pathway encode the same enzyme, most highly expressing genes were selected as representative data shown in the graph of Fig.1D; such genes are marked in the rightmost column. The "FPKM" values indicate the expression levels of each gene provided by NGS service in RNA-seq analysis. The "Mean ratio" values indicate the mean of the ratios of the expression levels of each gene in the stalk-destined cell to that in the spore-destined cell for three RNA-seq measurements.

Gene name	Protein name	1st (FPKM)		2nd (FPKM)		3rd (FPKM)		Mean ratio
		stalk-	spore-	stalk-	spore-	stalk-	spore-	
commd1	COMM domain-containing protein 1	7.80	5.88	1.80	2.31	7.09	3.48	1.38
commd10	COMM domain-containing protein 10	29.52	20.45	7.00	7.26	27.37	20.80	1.24
cupJ	ricin B lectin domain-containing protein	18.17	15.00	13.08	10.91	21.44	17.24	1.22
kcnma1	"calcium-activated BK potassium channel, alpha subunit"	1.99	1.65	0.97	0.72	2.27	2.37	1.17
fimA	actin bundling protein	164.51	111.70	169.48	152.79	171.06	193.43	1.16
patA	P-type ATPase	413.42	313.25	487.59	421.95	417.29	600.70	1.06
cnrF	RabGAP/TBC domain-containing protein	14.69	13.98	12.09	11.62	16.13	16.64	1.02
acpA	subunit of heterodimeric actin capping protein cap32/34	569.29	537.42	517.79	499.66	535.96	610.04	0.99
potA	"calcium-activated BK potassium channel, alpha subunit family protein"	1.39	1.28	1.62	1.72	1.75	1.87	0.99
commd7	COMM domain-containing protein 7	28.73	24.96	8.95	11.57	27.33	26.60	0.98
cupD	calcium up-regulated protein	7.05	8.35	2.48	2.20	6.26	11.94	0.83
cupF	calcium up-regulated protein	56.66	64.32	78.70	71.69	72.93	161.98	0.81
cupI	ricin B lectin domain-containing protein	99.36	136.92	548.15	473.33	85.69	181.28	0.79
cupB	calcium up-regulated protein	96.60	137.40	91.96	92.40	114.97	246.12	0.72
cupC	calcium up-regulated protein	56.07	75.52	60.05	59.75	57.85	143.13	0.72
cupH	ricin B lectin domain-containing protein	48.21	68.62	37.08	39.49	62.10	142.48	0.69
cupG	calcium up-regulated protein	56.42	82.97	44.89	47.43	57.08	152.92	0.67
cupA	calcium up-regulated protein	ND	ND	ND	ND	ND	ND	ND
commd2	COMM domain-containing protein 2	ND	ND	ND	ND	ND	ND	ND
commd3	COMM domain-containing protein 3	ND	ND	ND	ND	ND	ND	ND
commd8	COMM domain-containing protein 8	ND	ND	ND	ND	ND	ND	ND

Table S3. RNA-seq analysis results on the calcium-related genes

List of 21 genes annotated as “up-regulation in the presence of calcium” in the "gene description" section in dictyBase. The “FPKM” values indicate the expression levels of each gene provided by NGS service in RNA-seq analysis. The “Mean ratio” values indicate the mean of the ratios of the expression levels of each gene in the stalk-destined cell to that in the spore-destined cell for three RNA-seq measurements. ND indicates “not detected”, in which FPKM values are smaller than 0.01.

Gene name	Protein name	1st (FPKM)		2nd (FPKM)		3rd (FPKM)		Mean ratio
		stalk-	spore-	stalk-	spore-	stalk-	spore-	
carA-1	cAMP receptor 1	ND	ND	ND	ND	ND	ND	ND
carA-2	cAMP receptor 2	ND	ND	ND	ND	ND	ND	ND
mrps4	ribosomal protein S4, mitochondrial	ND	ND	ND	ND	ND	ND	ND
dia2	hypothetical protein DDB_G0291253	13.55	59.66	12.65	13.55	24.71	30.91	0.65
dia1	hypothetical protein DDB_G0285431	0.88	1.00	0.74	0.71	1.09	1.07	0.98
cafA	calfumirin-1	13.79	15.13	17.00	5.88	25.96	11.79	2.00
dscA-1	discoidin I, alpha chain	ND	ND	ND	ND	ND	ND	ND
dscA-2	discoidin I, alpha chain	ND	ND	ND	ND	ND	ND	ND
dscC-1	discoidin I, beta/gamma chain	ND	ND	ND	ND	ND	ND	ND
dscC-2	discoidin I, beta/gamma chain	ND	ND	ND	ND	ND	ND	ND
dscD-1	discoidin I, delta chain	ND	ND	ND	ND	ND	ND	ND
dscD-2	discoidin I, delta chain	ND	ND	ND	ND	ND	ND	ND
nxnA	annexin VII	1164.16	1073.03	1528.27	1418.91	1307.47	1607.23	0.99

Table S4. RNA-seq analysis results on the cell cycle-related genes

List of 13 genes related to cell cycle reported in previous studies (Maeda, 2005; Maeda, 2011). The "FPKM" values indicate the expression levels of each gene provided by NGS service in RNA-seq analysis. The "Mean ratio" values indicate the mean of the ratios of the expression levels of each gene in the stalk-destined cell to that in the spore-destined cell for three RNA-seq measurements. ND indicates "not detected", in which FPKM values are smaller than 0.01.

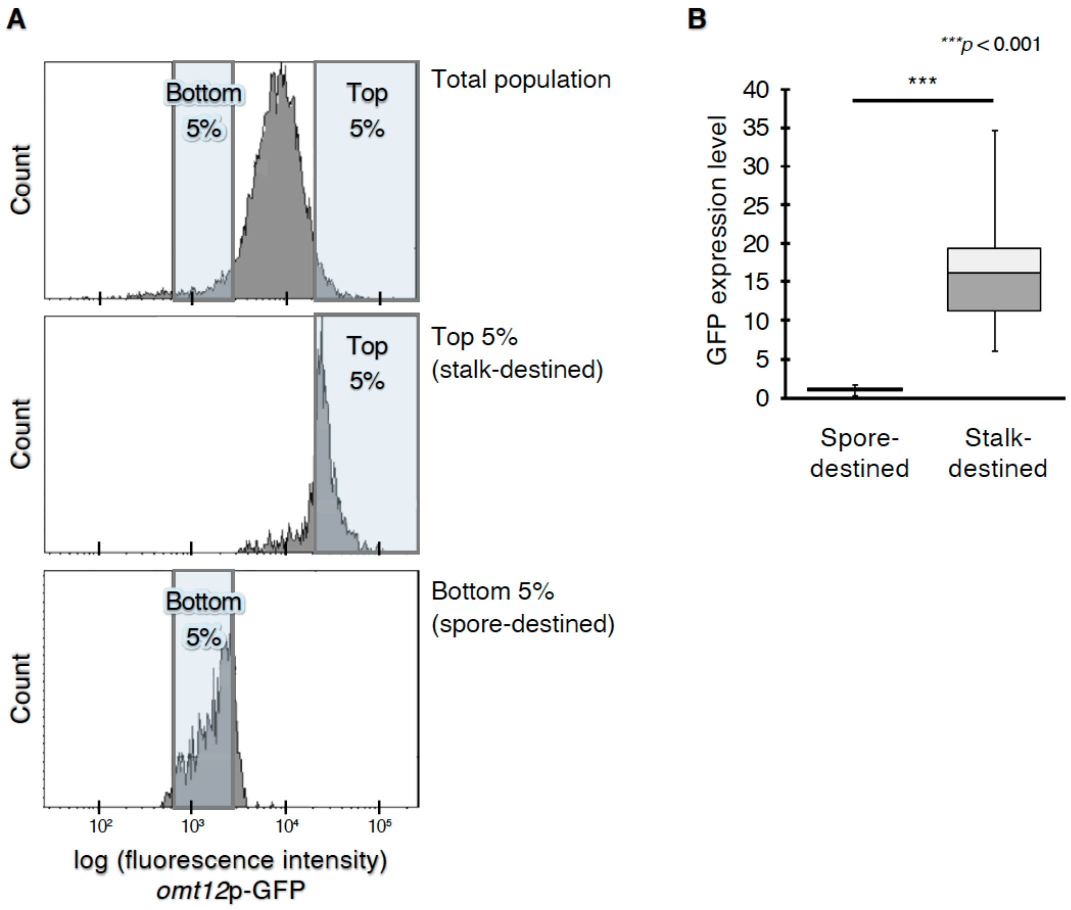

Figure S1. Quality-check of cell sorting.

(A) The fluorescence intensity of *omt12*p-GFP expressing cells (Kuwana et al., 2016) was measured by flow cytometry before and after sorting to quality-check the cell specimens. Typical fluorescence intensity distributions of total cell populations are shown: before sorting (top panel), after sorting the top 5% population (stalk-destined; middle panel), and after sorting the bottom 5% population (spore-destined; bottom panel).

(B) Expression levels of *omt12*p-GFP as measured by RT-PCR in spore-destined and stalk-destined cells sorted in (A). The average expression level of the spore-destined cells was set to 1. The results are presented as a box-and-whisker plot: the box indicates the median and the upper and lower quartiles; the whisker indicates the range. The numbers (n) of measurements: n = 29 (spore-destined) and n = 28 (stalk-destined). ***$p < 0.001$.

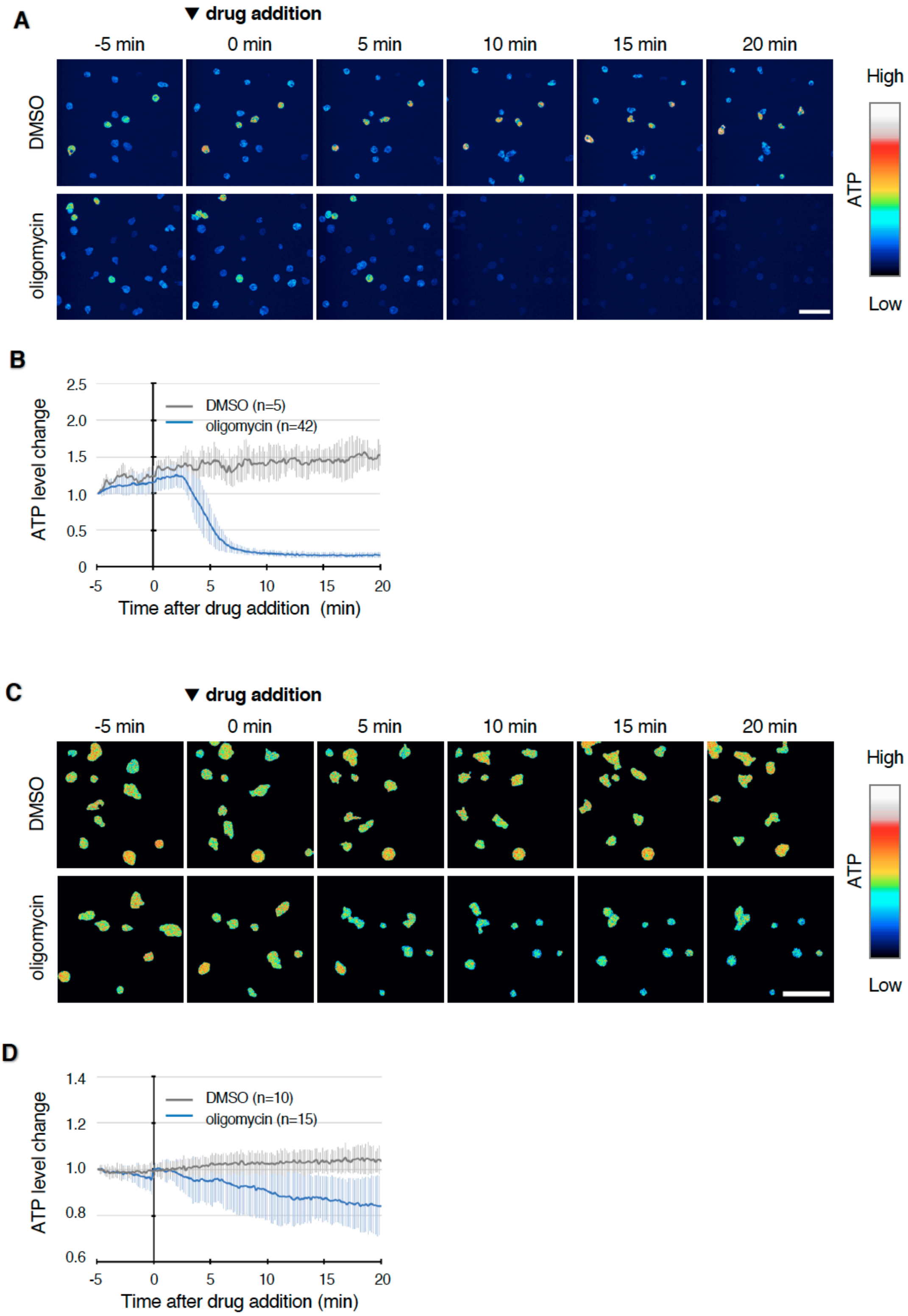
A
▼ drug addition
-5 min
0 min
5 min
10 min
15 min
20 min
DMSO
oligomycin
High
ATP
Low
B
ATP level change
DMSO (n=5)
oligomycin (n=42)
Time after drug addition (min)
C
▼ drug addition
-5 min
0 min
5 min
10 min
15 min
20 min
DMSO
oligomycin
High
ATP
Low
D
ATP level change
DMSO (n=10)
oligomycin (n=15)
Time after drug addition (min)

Figure S2. Evaluation of the ATP probes.

(A) Fluorescence images of DicMaLionG. The vegetative cells of the DicMaLionG expressing strain were treated with oligomycin (bottom panels), or dimethyl sulfoxide (DMSO) as a control solvent (top panels), for the indicated time periods. ATP levels are indicated by the color scale on the right. Images were acquired every 10 sec by Dragonfly200 using a 60× oil immersion objective lens. Scale bar, 50 µm.

(B) Changes in fluorescence intensities of DicMaLionG (ATP level) obtained in (A) were plotted over time during treatment with oligomycin or DMSO. Changes in fluorescence intensities were measured over time for individual cells by Fiji and plotted by setting the starting intensity (−5 min) to 1. Blue and gray lines indicate oligomycin and DMSO treatment, respectively. The number (n) of cells: n = 42 (oligomycin) and n = 5 (DMSO). The mean and SD are shown.

(C) FRET ratio images of DicAT1.03NL. The vegetative cells of the DicAT1.03NL expressing strain were treated with oligomycin (bottom panels), or DMSO as a control solvent (top panels), for the indicated time periods. ATP levels are indicated by the color scale on the right. These images are ratio images depicting the values of YFP/CFP intensity (see Materials and Methods section for details). Images were acquired every 10 sec by LSM780 using a 63× oil immersion objective lens. Scale bars, 50 µm.

(D) Changes in the YFP/CFP ratios of DicAT1.03NL (ATP level) obtained in (C) were plotted over time during treatment with oligomycin or DMSO. Changes in the YFP/CFP ratios were calculated over time for each cell and plotted by setting the starting intensity ratio (−5 min) to 1. Blue and gray lines indicate oligomycin and DMSO treatment, respectively. The number (n) of cells: n = 15 (oligomycin) and n = 10 (DMSO). The mean and SD are shown.

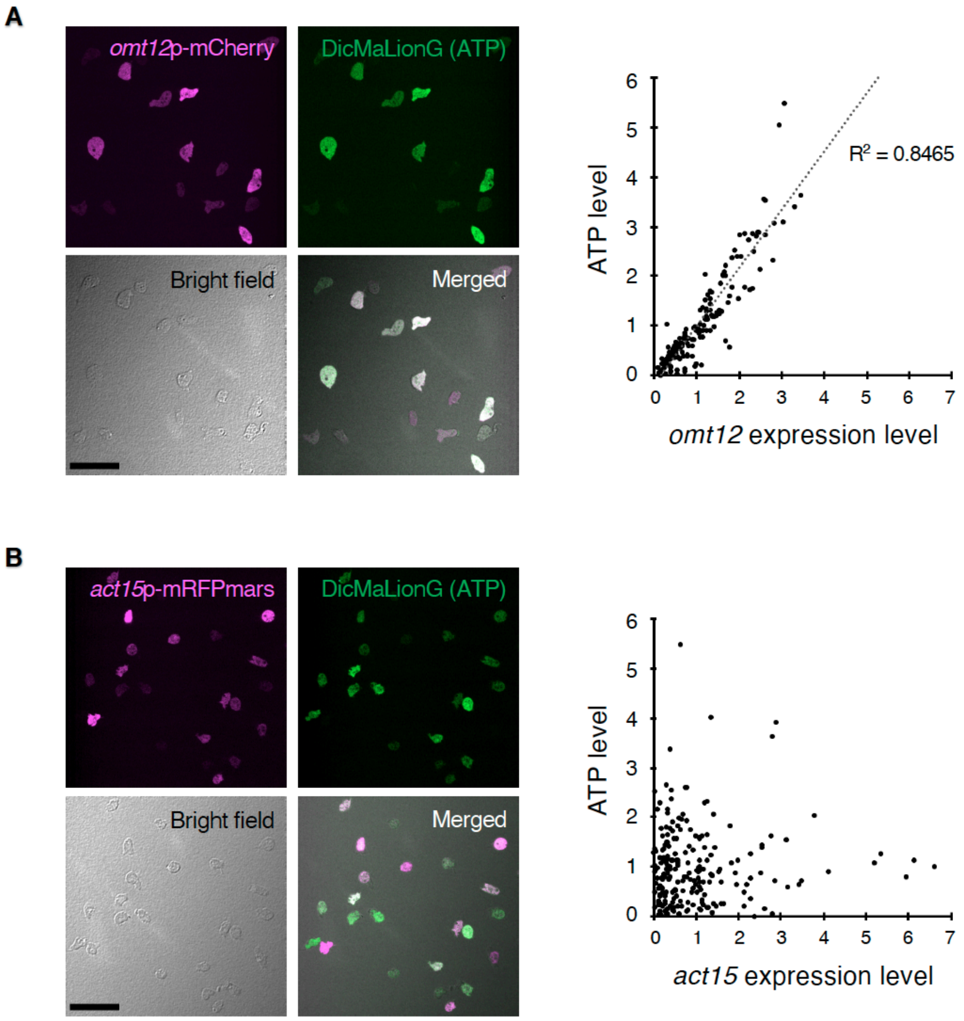

Figure S3. Correlation between *omt12*p-mCherry expression level and ATP level

(A) Images of cells of the *omt12*p-mCherry/DicMaLionG double-expressing strain: *omt12*p-mCherry (magenta), DicMaLionG (ATP) (green), a bright field image, and the merged image as indicated in the panels. These images were acquired by FV1000 using a 60× oil immersion objective lens. Scale bar, 50 µm. The graphs indicate plots of the values obtained by measuring the intensity of each cell and dividing by the mean intensity; the line indicates the linear regression of the plots. The number (n) of cells: n=190.

(B) Images of cells of the *act15*p-mRFPmars/DicMaLionG double-expressing strain: *act15*p-mRFPmars (magenta), DicMaLionG (ATP) (green), a bright field image, and the merged image as indicated in the panels. These images were acquired by FV1000 using a 60× oil immersion objective lens. Scale bar, 50 µm. The graphs indicate plots of the values obtained by measuring the intensity of each cell and dividing by the mean intensity. The number (n) of cells: n = 214.

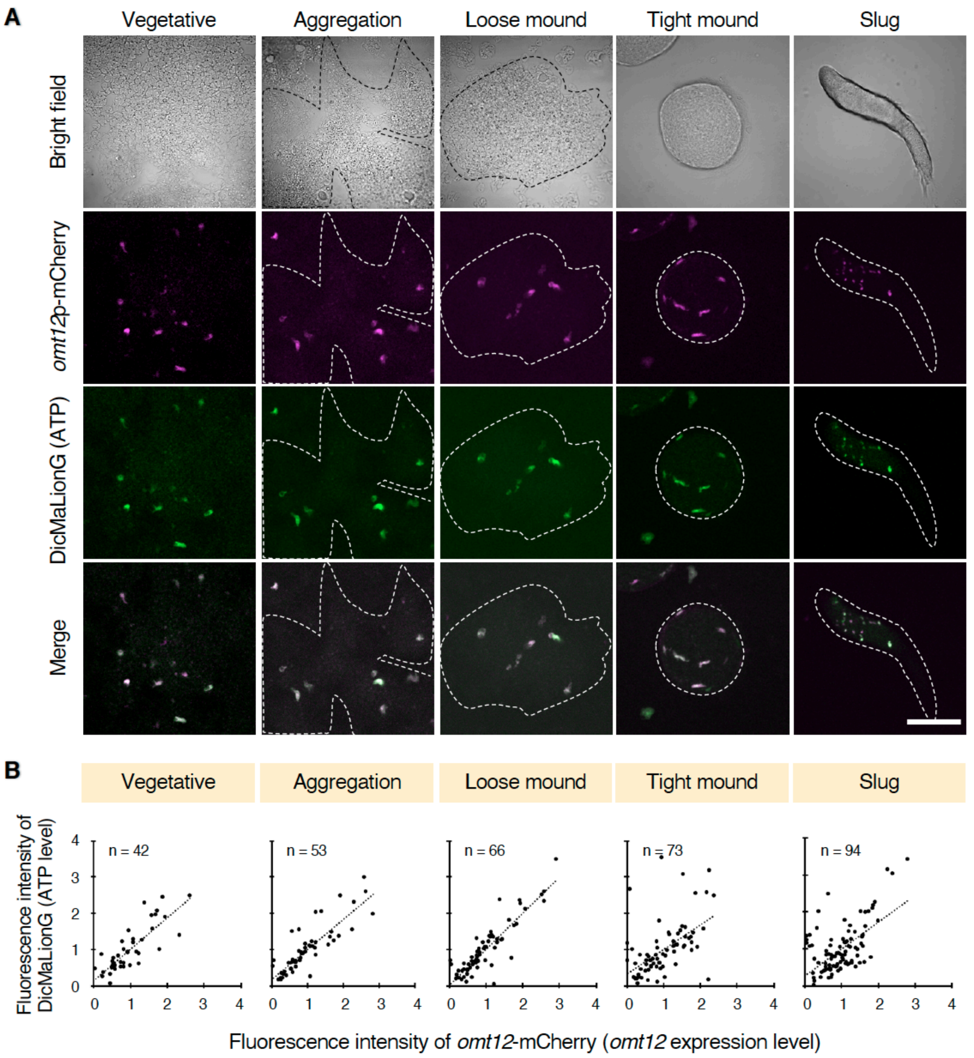

Figure S4. Correlation between *omt12*p-mCherry expression level and ATP level at various developmental stages

(A) Cells of the *omt12*p-mCherry/DicMaLionG double-expressing strain were mixed with wild-type Ax2 cells (not expressing fluorescent protein) at a ratio of 1%, and the process of development was observed. Bright-field images (top row), fluorescence images of *omt12*p-mCherry (magenta; second row), fluorescence images of DicMaLionG (ATP) (green; third row), and their merged images (bottom row) are shown. Dashed lines indicate the periphery of cellular population. These images were obtained by FV1000 using a 40× objective lens. Scale bars, 50 µm.

(B) Correlation between two fluorescent proteins. The graphs indicate plots of the values obtained by measuring the intensity of each cell and dividing by the mean intensity; the line indicates the linear regression of the plots. The number (n) of the cells is indicated in each graph.

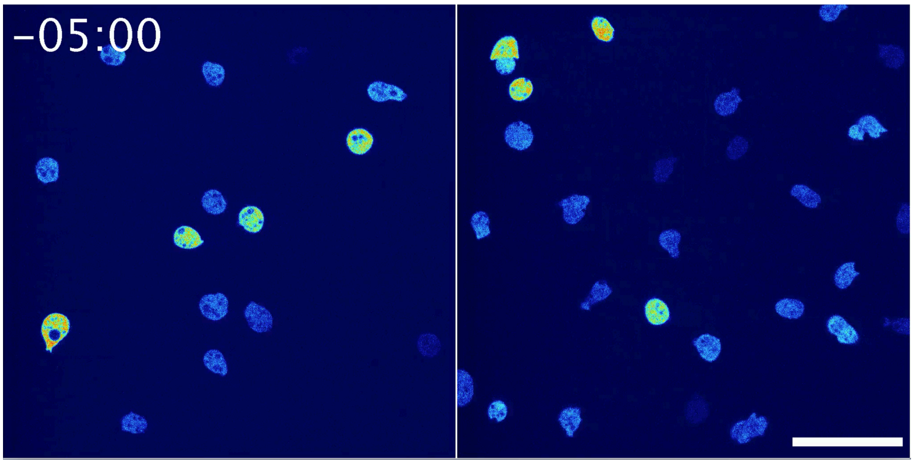

Movie S1. Evaluation of DicMaLionG as an ATP sensor probe

The movie observing changes in fluorescence intensities of DicMaLionG in cells treated with DMSO (left) or oligomycin (right). The images were acquired by Dragonfly200 using a 60× oil immersion objective lens by 10 sec intervals for 25 min. Their fluorescence intensities (ATP levels) are indicated by the color scale same as that mentioned in Fig. S2A. The number in the movie indicates time in minutes:seconds. DMSO or oligomycin (final concentration is 2.5 μM) was added at time 0. Scale bar, 50 μm.

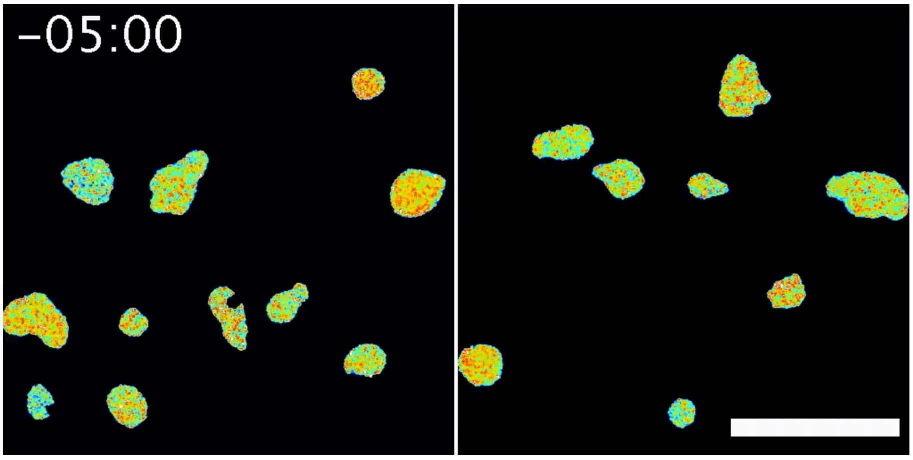

Movie S2. Evaluation of DicAT1.03NL as a FRET-type ATP sensor probe

The movie observing changes in CFP/YFP ratios of DicAT1.03NL in cells treated with DMSO (left) or oligomycin (right). The images were acquired by LSM780 using a 63× oil immersion objective lens by 10 sec intervals for 25 min. Their YFP/CFP ratios (ATP levels) are indicated by the color scale same as that mentioned in Fig. S2B. The number in the movie indicates time in minutes:seconds. DMSO or oligomycin (final concentration is 2.5 μM) was added at time 0. Scale bar, 50 μm.

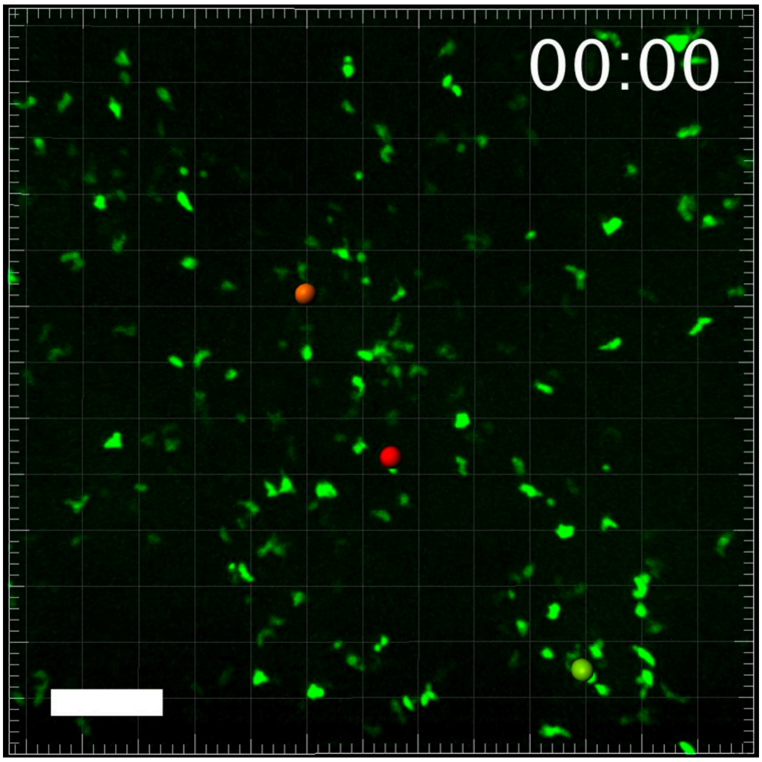

Movie S3. Tracking of cells expressing DicMaLionG during early development from the vegetative phase to the mound phase

Z-stack images were acquired every 3 min by Dragonfly200 using a 20× objective lens and projected with the maximum intensity using Fiji. Cell tracking was performed by Imaris software based on fluorescence intensities of DicMaLionG. The color of trajectories and spheres represents the fluorescence intensities (ATP level) at each time point by the same color scale in Fig. 3A (red is high, blue and green is low). The number in the movie indicates time in hours:minutes. Scale bar, 100 μm.

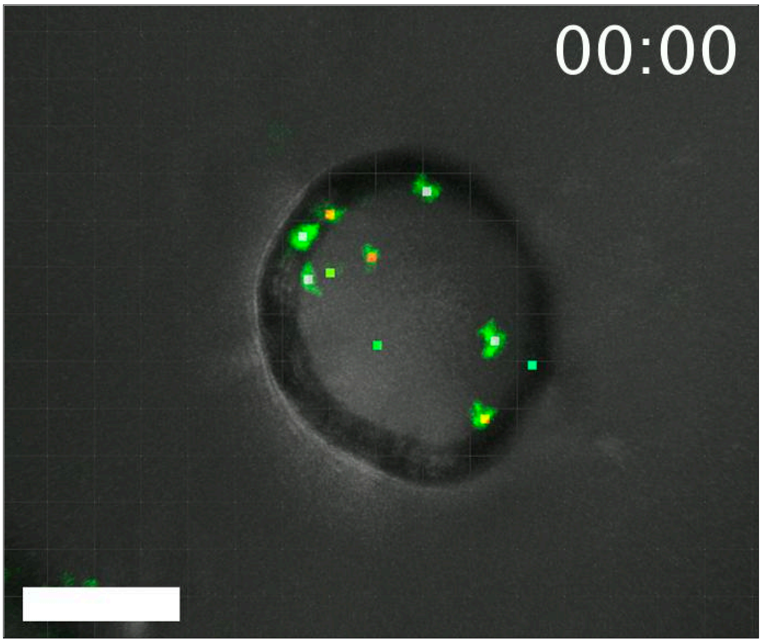

Movie S4. Tracking of cells expressing DicMaLionG during late development from the mound phase to the slug phase

Z-stack images were acquired every 3 min by Dragonfly200 using a 20× objective lens. Cell tracking was performed by Imaris software based on fluorescent intensity of DicMaLionG. The color of trajectories and spheres represents the fluorescent intensity (ATP level) at each time points by the same color scale in Fig. 3B (red is high, blue and green is low). Scale bar, 100 µm. The number in the movie indicates time in hours:minutes.

Data set S1. RNA-seq results of all genes tested

List of RNA-seq results of all tested genes in *D. discoideum*. Out of 12,257 protein-coding-genes, 8,191 genes were detected at the vegetative phase after two thresholding steps. The "FPKM" values and the "Count" values for the stalk-destined and spore-destined cells were provided by NGS service in three RNA-seq analyses. "FPKM" values, indicating expression levels of each gene, are calculated based on "Count" values and the base-pair length of each gene. The "Ratio (stalk/spore)" values indicate ratios of the "FPKM" values of each gene in the stalk-destined cells to that in the spore-destined cells. N/A in the "dictyBase ID" column indicates "not applicable", i.e., the genes are not identified in dictyBase.

Data set S2. RNA-seq results of metabolism-related genes

List of RNA-seq results of 304 metabolism-related genes selected based on annotation in dictyBase. Annotation of each gene is indicated in the left 3 columns: "M", metabolic process; "G", glycolytic process; "T", TCA cycle. The "FPKM" values and the "Count" values for the stalk-destined and spore-destined cells were provided by NGS service in three RNA-seq analyses. "FPKM" values, indicating the expression levels of each gene, are calculated based on "Count" values and the base-pair length of each gene. The "Ratio (stalk/spore)" values indicate ratios of the "FPKM" values of each gene in the stalk-destined cells to that in the spore-destined cells.
